# Supplementary material for: Adolescent stress exposure: behavioral consequences and molecular mechanisms in corticolimbic networks
Source: Front Cell Neurosci. 2026 Aug 6;20:1876565. doi: 10.3389/fncel.2026.1876565 (PMC13489889; doi:10.3389/fncel.2026.1876565)
Supplement: Supplementary file 1 [file Supplementary_file_1.docx]

**1. List and order of stressors used in each experiment**

**Experiments 1 and 2**

| **Day** | **Time of day** | **Stressor** |
| --- | --- | --- |
| 1 | AM | Shaker stress 1h 100 rpm |
|  | PM | Hypoxia 30 min |
| 2 | AM | Warm swim 20 min 31-33°C |
|  | PM | Shaker 1h 100 rpm |
|  | Overnight | Overnight Crowding |
| 3 | AM | Cold room 1h 4°C |
|  | PM | Cold swim 10 min 16-18°C |
| 4 | AM | Warm swim 20 min 31-33°C |
|  | PM | Shaker 1h 100 rpm |
| 5 | AM | Hypoxia 30 min |
|  | PM | Cold swim 10 min 16-18 °C |
|  | Overnight | Overnight isolation |
| 6 | AM | Shaker stress 1h 100 rpm |
|  | PM | Cold room 1h 4°C |
| 7 | AM | Warm swim 20 min 31-33°C |
|  | PM | Cold swim 10 min 16-18 °C |
| 8 | AM | Cold swim 10 min 16-18 °C |
|  | PM | Shaker 1h 100 rpm |
|  | Overnight | Overnight Crowding |
| 9 | AM | Hypoxia 30 min |
|  | PM | Cold room 1h 4°C |
| 10 | AM | Shaker stress 1h 100 rpm |
|  | PM | Warm swim 20 min 31-33°C |
|  | Overnight | Overnight isolation |
| 11 | AM | Cold swim 10 min 16-18 °C |
|  | PM | Cold room 1h 4°C |
| 12 | AM | Hypoxia 30 min |
|  | PM | Warm swim 20 min 31-33°C |
| 13 | AM | Shaker 1h 100 rpm |
|  | PM | Cold swim. 10 min 16-18 °C |
|  | Overnight | Overnight Crowding |
| 14 | AM | Warm swim 20 min 31-33°C |
|  | PM | Hypoxia 30 min |
| 15 | AM | Hypoxia 30 min |
|  | PM | Cold swim 10 min 16-18 °C |
| 15 | AM | Cold room 1h 4°C |
|  | PM | Hypoxia 30 min |
|  | Overnight | Overnight isolation |
| 17 | AM | Warm swim 20 min 31-33°C |
|  | PM | Shaker 1h 100 rpm |
| 18 | AM | Cold room 1h 4°C |
|  | PM | Shaker 1h 100 rpm |
| 19 | AM | Warm swim 20 min 31-33°C |
|  | PM | Cold room 1h 4°C |
|  | Overnight | Overnight Crowding |
| 20 | AM | Hypoxia 30 min |
|  | PM | Cold swim 10 min 16-18 °C |
| 21 | AM | Cold room 1h 4°C |
|  | PM | Hypoxia 30 min |

**Experiments 3 to 5**

| **Day** | **Time of day** | **Stressor** |
| --- | --- | --- |
| 1 | AM | Shaker stress 1h 100 rpm |
|  | PM | Hypoxia 30 min |
| 2 | AM | Cold room 1h 4°C |
|  | PM | Warm swim 20 min 31-33°C |
|  | Overnight | Overnight Crowding |
| 3 | AM | Cold room 1h 4°C |
|  | PM | Restraint 30 min |
| 4 | AM | Cold swim 10 min 16-18 °C |
|  | PM | Shaker 1h 100 rpm |
| 5 | AM | Hypoxia 30 min |
|  | PM | Restraint 30 min |
|  | Overnight | Overnight isolation |
| 6 | AM | Cold room 1h 4°C |
|  | PM | Shaker 1h 100 rpm |
| 7 | AM | Warm swim 20 min 31-33°C |
|  | PM | Restraint 30 min |
| 8 | AM | Warm swim 20 min 31-33°C |
|  | PM | Cold swim 10 min 16-18 °C |
|  | Overnight | Overnight Crowding |
| 9 | AM | Hypoxia 30 min |
|  | PM | Cold room 1h 4°C |
| 10 | AM | Cold room 1h 4°C |
|  | PM | Shaker stress 1h 100 rpm |
|  | Overnight | Overnight isolation |
| 11 | AM | Restraint 30 min |
|  | PM | Warm swim 20 min 31-33°C |
| 12 | AM | Hypoxia 30 min |
|  | PM | Cold swim 10 min 16-18 °C |
| 13 | AM | Shaker 1h 100 rpm |
|  | PM | Restraint 30 min |
|  | Overnight | Overnight Crowding |
| 14 | AM | Cold swim 10 min 16-18 °C |
|  | PM | Hypoxia 30 min |

**2. List of genes quantified and manufacture’s probe codes used in each case:**

1. nr3c1, Glucocorticoid receptor, GR (Rn00561369_m1);
2. nr3c2, Mineralocorticoid receptor, MR (Rn00565562_m1);
3. Adcyap1, adenylate cyclase activating polypeptide 1,PACAP (Rn00566438_m1);
4. Adcyap1r1, adenylate cyclase activating polypeptide 1 receptor 1, Pac1 (Rn01763128_m1);
5. VIP, vasoactive intestinal polypeptide VIP (Rn01430567_m1);
6. Fkbp5, FK506 binding protein 5, Fkbp51 (Rn01768371_m1);
7. Adra2a, adrenergic receptor, alpha 2a (Rn00562488_s1);
8. EGR1, early growth response 1 (Rn00561138_m1) ;
9. CRH, corticotrophin releasing hormone (Rn01462137_m1);
10. Adra1d, adrenergic receptor, alpha 1d (Rn00577931_m1);
11. Adra1b, adrenergic receptor, alpha 1b (Rn01471343_m1);
12. Adrb1, adrenergic receptor, beta 1 (Rn00824536_s1);
13. BDNF, brain derived neurotrophic factor (Rn02531967_s1);
14. Gabra1, gamma-aminobutyric acid (GABA) A receptor, subunit alpha 1 (Rn00788315_m1);
15. Gabrg1, gamma-aminobutyric acid (GABA) A receptor, subunit gamma 1 (Rn00589841_m1);
16. Gabbr2, gamma-aminobutyric acid (GABA) B receptor, 2 ( Rn00582550_m1);
17. Htr2a, 5-hydroxytryptamine (serotonin) receptor 2A (Rn00568473_m1);
18. Htr1b, 5-hydroxytryptamine (serotonin) receptor 1B (Rn01637747_s1);
19. Penk, Preproenkephalin (Rn00567566_m1);
20. Slc6a11, solute carrier family 6 member 11, GABA transporter, Gat3 (Rn00577664_m1);

**Houskeeping genes**

1. B-actin , Rn00667869_m1
2. HPRT1, Rn01527840_m1
3. 18s rRNA, Hs99999901_s1

**3. 24-hour homecage activity**

14 days after the SDWS, animals were assesses for 24 h to determine their level of activity durring the circadian cycle. There was no efect of sex in each sex

**4.Supplmental figures Morris water maze**

**A-B:** Every session and trial within session during the 4-day training paradigm.**C-g:** Latency to enter probe quadrant during the probe test. **D-H:** Number of entries to the probe quadrant during the probe test. **E-I:** Total distance covered during probe test. **F-K:** Average velocity during probe test. No significant effects observed in any of these

**5- Habituation phases of the novel object recognition test.** Before testing for novel object recognition, animals were habituated to the familiar object twice, a day before the test and on the test day, 1 hour before one of the objects was replaced by the novel object. Rats did not show any previous bias towards one of the sides. The location (left or right) for the novel object in the following session was conterbalanced between anaimals.

**6. Habituation phase social 3.-chamber test.** Before testing, the first session consisted of habituating the animals to the apparatus in the presence of the empty carrousels. Rats spent equal time in all the chambers.
